# Exploring the interconnections of loneliness, anxiety, and depression among nursing students: a network analysis approach

**DOI:** 10.3389/fpsyt.2025.1537935

**Published:** 2025-02-17

**Authors:** Yuqing Mi, Sukhee Ahn, Liping Ren

**Affiliations:** ^1^ College of Public Health, Shandong Second Medical University, Weifang, China; ^2^ College of Nursing, Chungnam National University, Daejeon, Republic of Korea; ^3^ College of Nursing, Shandong Second Medical University, Weifang, China

**Keywords:** anxiety, depression, loneliness, mental health symptomology, psychological networks, students, nursing, targeted interventions

## Abstract

**Objective:**

Nursing students experience higher rates of anxiety and depression than students in other disciplines due to the demanding academic requirements and clinical training challenges. Loneliness and symptoms of anxiety and depression occur simultaneously; however, the specific interrelationships between these states remain inadequately investigated. This study aimed to utilize network analysis to examine the item-level reciprocal action between loneliness, anxiety, and depression among nursing students.

**Methods:**

A total of 888 nursing students were assessed using the short-form UCLA Loneliness Scale (ULS-6), the Generalized Anxiety Disorder 7-item Questionnaire (GAD-7), and the Patient Health Questionnaire-9 (PHQ-9) on loneliness, anxiety, and depression, respectively. Descriptive analyses were conducted using SPSS 26.0, whereas other statistical analytical procedures were performed using R software. The Gaussian graphical model was used to estimate network, and the Network Comparison Test was applied to compare differences in networks across gender and grades.

**Results:**

The results indicated that 58.6% of nursing students exhibited varying degrees of loneliness. Network analysis revealed that loneliness formed a separate cluster with limited connections to anxiety and depression communities. The edges between PHQ3-PHQ4 (insomnia or hypersomnia and fatigue), GAD1-GAD2 (feeling anxious and excessive worrying), ULS1-ULS2 (lonely and no one) showed the strongest positive edges within their communities, respectively. And the strongest inter-community edges were observed between GAD5-PHQ8 (fidgety-retardation), ULS6-PHQ4 (isolation-fatigue), and ULS1-GAD1 (lonely-feeling anxious). The centrality analysis identified GAD2 (excessive worrying), ULS6 (isolation), PHQ4 (fatigue), and PHQ2 (feeling down) as the most central node, indicating their significant influence on the overall network structure. Additionally, PHQ8 (retardation), PHQ2 (feeling down), GAD5 (fidgety), and GAD1(feeling anxious) played a crucial role as bridging symptoms that linked the three communities. In addition, there is no statistically significant difference in the network structure except strength of GAD3 (generalized anxiety) and GAD6 (irritable) between sexes.

**Conclusions:**

This study highlights the high prevalence of loneliness among nursing students and its distinct yet limited connection to anxiety and depression, emphasizing its unique role as a standalone psychological construct. The central symptoms in the network and important bridge symptoms across different psychological communities highlight the complexity of mental health symptom networks. This underscores the importance of targeting central symptoms for domain-specific interventions and addressing bridge symptoms to mitigate comorbidities across psychological conditions among nursing students.

## 1 Introduction

One global concern is the poor mental health of university students (1). Anxiety and depression are prevalent mental disorders that frequently co-occur and often precipitate each other (2). Studies of anxiety symptom prevalence have estimated such at 15.8%–61.0%, while depression prevalence is 22.3%–69.5% among university students; although this figure varies across different countries (3, 4), factors such as gender, excessive academic pressure (5), unhealthy behaviors, traumatic experiences, family problems (1), and poor interpersonal relationships (6) can lead to depression and anxiety. Numerous recent studies have demonstrated that nursing students have higher levels of anxiety and depression than students in alternative academic fields (1), primarily because nursing programs with rigorous academic requirements and coping issues for clinical training present unique challenges compared to other undergraduate programs (7). Understanding the mechanisms that contribute to an increased risk of anxiety and depression among nursing students is important for developing coping strategies and targeted interventions aimed at preventing and alleviating distress.

Loneliness is recognized as a public mental health concern and previous studies have indicated its correlation with the development of depression and other prevalent mental health issues (8). Bahr defines loneliness as a state in which individuals perceive a lack of intimacy and social connection (9). A survey in Germany showed that the current loneliness of university students was more serious: 3.2% of university students said that they felt severely lonely, with 32.4% reporting feeling moderately lonely (10). The link between loneliness, anxiety, and depression has recently received considerable attention (7, 11). Based on these studies, a complicated and interdependent relationship exists between poor mental health and loneliness (12), and young adults who experience loneliness are more likely to experience mental health problems (13). Research has pointed to the reciprocal effect of loneliness on anxiety and depressive symptoms (14). These results reveal a connection between the symptoms of depression, anxiety, and loneliness. Afzali highlighted the complex interconnections that exist between symptoms, which may show up as an interconnected network of symptoms (15). However, it is unclear how loneliness is related to depression and anxiety among nursing students and research is relatively sporadic. Therefore, it is necessary to understand the connection between loneliness and the symptoms of anxiety and depression to provide effective interventions to alleviate psychological problems among nursing students.

Network analysis is an integrative approach to the structure of psychopathology that can analyze the signs of complications arising from two or more disorders/syndromes and display complex relationships between variables in an intuitive way by conceptualizing the structure of the variables of study as a network of interconnected symptomatic nodes whose edges indicate the association between two nodes controlling other variables in the network (16). Unlike traditional methods, it is useful to identify central symptoms related to other symptoms and bridging symptoms that connect two disorders and may increase the likelihood of symptomatic transfer from one disorder to another (17, 18). From this perspective, the emergence of loneliness, anxiety, and depression is understood as the result of complex interactions between individual symptoms (nodes) rather than being attributed to a single, overarching underlying cause (19). Accurately depicting these interactions is crucial for understanding the psychopathological mechanisms underlying these conditions and for developing targeted intervention strategies (19, 20). Thus, network analysis is a suitable technique for investigating the complex connections between depression, anxiety, and loneliness.

Previous studies have extensively explored the network of symptoms involving loneliness, anxiety, and depression across different populations including, highlighting the intricate relationships among these domains (11, 19, 21, 22). Central symptoms such as depression-related “fatigue” and anxiety-related “irritability,” and bridge symptoms like “depressed mood” being consistently identified in anxiety-depression network among college students across different disciplines (7, 23, 24). However, findings on the role of loneliness in these networks remain inconsistent. For example, loneliness has been found to be the most central node in network analysis research on depression, indicating that loneliness is most tightly linked to other symptoms of depression among adolescents (22). According to a Chinese study, one bridge symptom of loneliness (people around me but not with me) exhibited a greatly enhanced effect on two network structures of loneliness-anxiety and loneliness-depression, respectively (19). In contrast, a Polish study showed that loneliness had the lowest centrality of all the nodes in a network of adolescent symptoms of depression (21). Similarly, Owczarek investigated the correlation between loneliness, anxiety, and depression based on a population network, and their findings contradicted the theory that treating loneliness would help people feel less anxious or depressed (11). These discrepancies suggest that the association between loneliness, anxiety, and depression may be influenced by factors such as age, culture, particular stressors, and history of disease (25–27). Thus, it is essential to examine the complex link between loneliness and the symptoms of anxiety and depression in particular populations. Despite the growing body of research, limited studies have focused on nursing students, a group particularly vulnerable to loneliness, anxiety, and depression due to their unique academic and clinical stressors (1, 7). Therefore, exploring the complex links of loneliness with anxiety and depression in this specific population is essential for providing concrete evidence and effective interventions to alleviate their loneliness, anxiety, and depression.

This study utilized network analysis to explore the complex relationships between loneliness, anxiety, and depression among Chinese nursing students. Emphasis was placed on the identification of central and bridge symptoms within the network model. The findings of this study will contribute to a deeper understanding of the correlations among loneliness, anxiety, and depression, providing targeted evidence and interventions to boost nursing students’ mental health.

## 2 Methods

### 2.1 Design and participants

A cross-sectional descriptive survey was conducted to construct and evaluate a network model of loneliness, anxiety, and depression among Chinese nursing students. A convenience sample consisting of 888 participants from two universities in XX province, China, was recruited from May to September 2023. Nursing students were enrolled in this study if they (a) could speak Chinese, (b) were undergraduate nursing students, and (c) agreed to participate in the survey with written consent. Nursing students who had participated in similar research within the past three months were excluded from the study. However, 181 incomplete responses (16.9%) were excluded, resulting in an 83.1% effective response rate.

The sample size was determined based on recommendations from the literature on network analysis. Epskamp et al. suggest that 500 or more participants are sufficient for partial correlation network analysis using Gaussian Graphical Models (28). Additionally, Constantin and Cramer conducted a simulation study to evaluate the impact of various design factors on network estimation. For networks with 20 nodes, their findings indicate that 200–500 participants are required for sparse networks, 550 for moderately dense networks, and 600 for dense networks (29). Therefore, a sample size of 888 participants was considered appropriate for this study.

### 2.2 Data collection

The data for this study was gathered through a self-reported questionnaire using a secure web-based survey platform (www.wjx.cn) to ensure anonymity. The survey was prefaced with an introductory letter explaining the study’s aims, significance, and confidentiality policies to inform and reassure the participants. Nursing students were invited to participate by the university’s School of Nursing counselors, who distributed the survey link via WeChat and provided access through QR code scanning. To ensure a diverse and representative sample, students from all academic years were invited, and participation was entirely voluntary. Measures were taken to minimize potential biases introduced by convenience sampling, including broad dissemination of the survey and clear instructions to all participants. To ensure rigor and consistency in data collection, the principal investigator thoroughly trained two research assistants during the survey administration process. The assistants monitored the quality of the responses and addressed any issues. As a token of appreciation and encouragement, each nursing student who completed the survey received a small monetary incentive of 5 yuan. To address potential ethical concerns, the incentive amount was kept minimal to avoid coercion, and participation was entirely voluntary, with students informed they could withdraw at any time without penalty. The study was approved by the Ethics Committee, ensuring all ethical guidelines were followed.

### 2.3 Measures

#### 2.3.1 Loneliness

Loneliness was evaluated using the Chinese version of the short-form UCLA Loneliness Scale (ULS-6) (30), which contains six items (items 3 and 6 were reverse-scored). Each item is rated on a 4-point Likert scale ranging from 1 (never) to 4 (always), and the total score ranges from 6 to 24 points, with higher scores suggesting a higher level of loneliness. The Cronbach’s α was.834 in the present study.

#### 2.3.2 Anxiety symptoms

The Generalized Anxiety Disorder 7-item Questionnaire (GAD-7) was used to measure anxiety symptoms (31). The GAD-7 has seven items, each with scores ranging from 0 (not at all) to 3 (nearly every day) and total scores ranging from 0 to 21, with higher scores indicating more severe anxiety. The Cronbach’s α was.927 in the present study.

#### 2.3.3 Depression symptoms

Depression symptoms was diagnosed by Patient Health Questionnaire-9 (PHQ-9), which published by the American Psychiatric Association (32). The scale includes 9 items, and each item is divided into four levels (0 ~ 3 points), each item is scored from 0 (“not at all”) to 3 (“almost every day”). The total score on this scale ranged from 0 to 27, the higher the total score, the more severe the depression. The Cronbach’s α of the scale was.891 in this study.

#### 2.3.4 Demographic characteristics

Demographic characteristics such as age, sex, and grade of nursing students were collected.

### 2.4 Statistical analysis

Descriptive analysis was conducted using SPSS version 26.0, whereas other statistical analytical procedures were performed using R software (version 4.1.3). The network was estimated using a Gaussian graphical model (33), an undirected network that calculates pairwise associations between symptoms through partial correlation analysis, controlling for all other variables in the network. The regularization of the Gaussian graphical model was performed by the Extended Bayesian Information Criterion (EBIC) Graphical Least Absolute Shrinkage and Selection Operator (LASSO) algorithm (34). In this regularization process, a more robust and interpretable sparse network was obtained by shrinking all edges and setting the edges with less bias correlation to zero (30). In addition, bridge network was plotted by defining bridge network variables on top of the plotted network. Predictability, which reflects the proportion of variance in a node explained by its neighboring nodes, was calculated using the R package *mgm* (version 1.2-14) (35). Network construction and visualization were performed using the R package *qgraph* (version 1.9.8) (36), where blue edges indicate positive correlations and red edges indicate negative correlations, with edge thickness representing the strength of the correlation.

To explore the central symptoms in the network, four centrality indices were calculated: strength, closeness, betweenness, and expected influence (36). The bridging function of R-package *networktools* (version 1.5.2) was used to explore bridging symptoms. The bridge centrality indices used in this study include bridge strength, bridge closeness, bridge betweenness, and expected bridge influence (18). Correlation stability (CS) coefficients, accuracy of the edge weights (37, 38), and bootstrapped difference tests were conducted using R-package *Boonet* (version 1.6) packages to assess the stability and accuracy of the network model. This study compared differences in networks across genders using the Network Comparison Test (NCT, version 2.2.2) package of R software (37, 39), a more detailed description of the analytical approach is displayed in **Supplementary Table 1**.

### 2.5 Ethical considerations

Data confidentiality was maintained, and the study was approved by the Ethics Committee of XX University on Jan 6, 2023 (Approval no. 2023XXXX2).

## 3 Results

### 3.1 Characteristics of participants

In total, 888 undergraduate nursing students met the inclusion criteria and completed the study. The mean age of the sample was 20.26 ± 1.25 years (range: 18 to 24 years). This included 169 freshmen (19.0%), 209 sophomores (23.5%), 424 juniors (47.7%), and 86 seniors (9.7%). Most participants were female (n = 725, 81.6%). **Table 1** illustrates the abbreviations, mean scores, standard deviations, and predictability of all items in the network.

**Table 1:** Mean scores, standard deviations, abbreviation and predictability for each node of the loneliness, anxiety and depression symptoms.

Nodes	Abbreviation	M	SD	Pr (%)
Loneliness (ULS-6)				
ULS1: Lack companionship	Lonely	1.82	.83	46.5
ULS2: No one I can turn to	No one	1.63	.84	30.9
ULS3: Feeling left out	Excluded	1.89	.78	47.0
ULS4: Unhappy being so withdrawn	Discontented	1.80	.83	29.4
ULS5: People are around me but not with me	Alienated	1.58	.72	42.6
ULS6: Feeling isolation from others	Isolation	1.92	.82	55.1
Anxiety symptoms (GAD-7)				
GAD1: Feeling nervous, anxious or on edge	Feeling anxious	.33	.55	32.6
GAD2: Not being able to stop or control worrying	Excessive worrying	.25	.51	30.6
GAD3: Worrying too much about different things	Generalized anxiety	.28	.51	23.0
GAD4: Trouble relaxing	Trouble relaxing	.30	.56	23.3
GAD5: Being so restless that it is hard to sit still	Fidgety	.16	.43	37.8
GAD6: Becoming easily annoyed or irritable	Irritable	.27	.51	29.8
GAD7: Feeling afraid as if something awful might happen	Apprehensive	.17	.44	31.1
Depression symptoms (PHQ-9)				
PHQ1: Little interest or pleasure in doing things	Anhedonia	.39	.61	27.8
PHQ2: Feeling down, depressed, or hopeless	Feeling down	.28	.51	39.0
PHQ3: Sleep difficulties	Insomnia or hypersomnia	.39	.67	4.4
PHQ4: Feeling tired or having little energy	Fatigue	.48	.65	32.1
PHQ5: Poor appetite or overeating	Disordered eating	.37	.64	20.4
PHQ6: Feeling bad about yourself	Low self-esteem	.23	.51	28.6
PHQ7: Trouble concentrating on things	Concentration difficulties	.31	.58	29.6
PHQ8: Psychomotor agitation/retardation	Retardation	.16	.42	39.7
PHQ9: Thoughts of death	Suicidal ideation	.09	.35	30.1

ULS, the short-form UCLA Loneliness Scale; GAD, The Generalized Anxiety Disorder 7-item Questionnaire; PHQ, Patient Health Questionnaire-9; M, mean; SD, Standard deviation; Pre, Predictability.

### 3.2 Descriptive statistics and relationships of main variables

The findings revealed that 58.6% of nursing students exhibited varying degrees of loneliness, ranging from mild (35.2%) and moderate (20.4%) to intense (2.9%) feelings of loneliness. Positive correlations were found between the score for loneliness and the scores for anxiety (r = .484, p <.001) and depression (r = .531 p <.001). Additionally, the score for anxiety was positively correlated with the score for depression (r =.783, p <.001).

### 3.3 Network analysis

#### 3.3.1 Network structure

**Figure 1:**
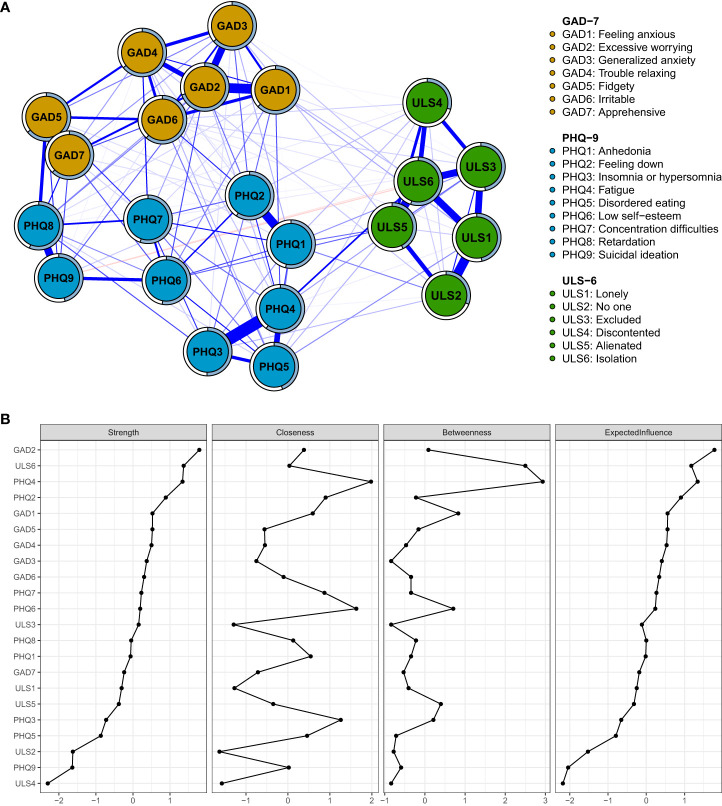
**(A)** Network structure of loneliness, depression, and anxiety. **(B)** Centrality Indices of all symptoms within the network. The thickness of an edge indicates the strength of the correlation. Blue lines represent positive connections, and red lines represent negative connections. The blue ring shows the proportion of explained variance. ULS, The short-form UCLA Loneliness Scale; GAD, The Generalized Anxiety Disorder 7-item Questionnaire; PHQ, Patient Health Questionnaire-9.

**Figure 1A** shows that the depression and anxiety communities were closely linked, whereas the loneliness community exhibited marginal associations with the depression the anxiety communities. Of the 231 edges, 133 (57.6%) were estimated to be nonzero. Node predictability is the variance explained by its neighboring symptoms, indicating the degree of local interconnection. And the range of values for node predictability in this study was 29.4% to 69.7%, with a mean value of 53.5%, indicating that 53.5% of the node variance in the current network could be explained by its neighbors (**Figure 1A**; **Table 1**). All edge weights within the loneliness, anxiety, and depression communities are presented in **Supplementary Table 2**. The strongest intra-community edges were identified as PHQ3-PHQ4 (insomnia or hypersomnia and fatigue, edge weight = .32), GAD1-GAD2 (feeling anxious and excessive worrying, edge weight = .28), and ULS1-ULS2 (lonely and no one, edge weight = .27). In comparison, the strongest inter-community edges were observed between GAD5-PHQ8 (fidgety-retardation, edge weight = .16), ULS6-PHQ4 (isolation-fatigue, edge weight = .12), and ULS1-GAD1 (lonely-feeling anxious, edge weight = .04). These findings indicate that inter-community edges were weaker than intra-community edges, reflecting the stronger connections within symptom clusters. Furthermore, the bootstrapped difference test for edge weights showed that, except for ULS1-GAD1 (lonely-feeling anxious), the key intra- and inter-community edges exhibited statistically significant differences in their weights (**Supplementary Figure 1**).

#### 3.3.2 Central symptoms

In the whole network, centrality indices measure a comprehensive understanding of a node’s role within the network, as demonstrated in **Figure 1B**; **Supplementary Table 3**, the symptom GAD2 (excessive worrying) with strength of 1.16 was the most central node, followed by ULS6 (isolation, strength = 1.10), PHQ4 (fatigue, strength = 1.10) and PHQ2 (feeling down, strength = 1.04), suggesting these symptoms have significant influence on the overall network structure.

#### 3.3.3 Bridge symptoms

**Figure 2:**
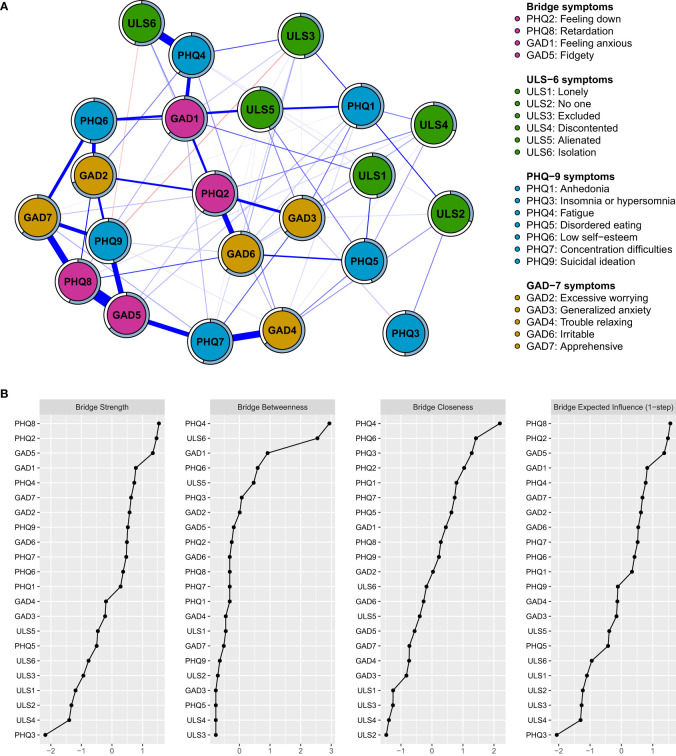
**(A)** Network structure of loneliness, depression and anxiety showing only bridge connections. **(B)** Bridge Centrality Index of all symptoms within the network. The thickness of an edge indicates the strength of the correlation. Blue lines represent positive connections, and red lines represent negative connections. The blue ring shows the proportion of explained variance. ULS, the short-form UCLA Loneliness Scale; GAD, The Generalized Anxiety Disorder 7-item Questionnaire; PHQ, Patient Health Questionnaire-9.

Bridge centrality refers to the role of a node in connecting different communities within a network, as showed in **Figure 2**; **Supplementary Table 2**, revealed that PHQ8 (retardation) with bridge strength of.40, PHQ2 (feeling down, bridge strength = .39), GAD5 (fidgety, bridge strength = .38), and GAD1 (feeling anxious, bridge strength = .36) acted as important bridges across loneliness, depression, and anxiety communities. These findings underscore the importance of recognizing bridging symptoms to highlight crucial transdiagnostic symptoms that can be targeted for the treatment of co-occurring conditions.

#### 3.3.4 Network stability and accuracy

**Figure 3:**
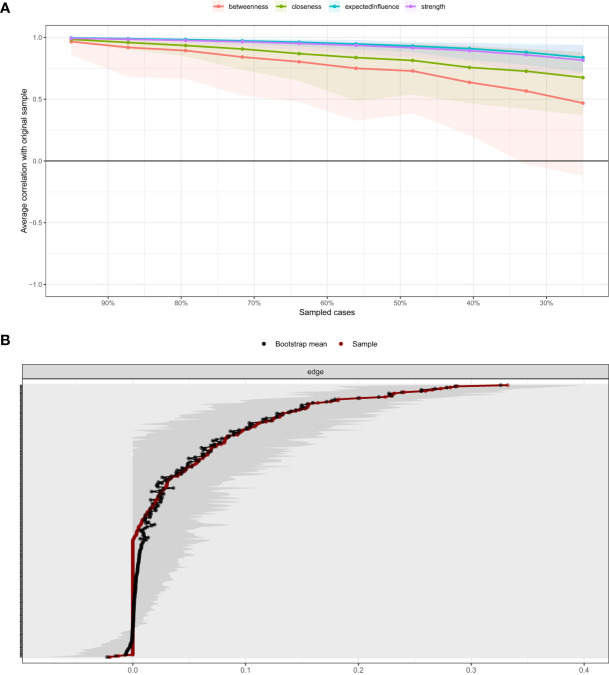
**(A)** Stability of centrality indices. The X-axis represents the proportion of sampled case at each step, while the Y-axis represents the mean correlations between the original indices and the subset indices. Colorful areas represent 95% CI. **(B)** Bootstrapped 95% CIs of estimated edge weight. The red line depicts the sample edge weights and the gray bar depicts the bootstrapped confidence interval. CI, confidence interval.

**Figure 3A** shows the case-dropping subset bootstrapping test. The findings indicated that, following the exclusion of 70% of the sample, the CS-coefficients for betweenness, bridge strength, closeness, expected influence, and strength exceeded.5, with CS-coefficients for strength and bridge strength measuring at.67 and.75 (all >.25), suggesting a high level of robustness in the network model. **Figure 3B** shows that the bootstrapped 95% confidence interval (CI) for the estimated edge weights was relatively narrow, indicating that the estimates were precise. The strength of the nodes GAD2 (excessive worrying), ULS6 (isolation), PHQ4 (fatigue), and PHQ2 (feeling down) were significantly different from other nodes in the network (**Supplementary Figure 2A**). The bridge strengths of the nodes PHQ8 (retardation), PHQ2 (feeling down), GAD5 (fidgety), and GAD1 (feeling anxious) were significantly more concentrated than the other nodes in the network (**Supplementary Figure 2B**).

#### 3.3.5 Comparative network analysis between sexes

The results of the comparative analysis of networks between sexes showed no significant difference between the networks of males and females (p = .109). Global strength invariance was not statistically significant (p = .851). In addition, there is a statistically significant difference in the strength of GAD3 (generalized anxiety, male:.86 and female: 1.02, p = .049), and GAD6 (irritable, male: 1.25 and female:.96, p = .010) of anxiety between sexes.

## 4 Discussion

This study revealed nursing students experience a high level of loneliness, and loneliness formed a separate cluster with limited connections to the anxiety and depression communities in the observed network model. Central symptoms, such as GAD2 (excessive worrying), PHQ4 (fatigue) and PHQ2 (feeling down), were most influential symptoms driving communities, and some depressive and anxiety symptoms (e.g., “retardation”, “feeling down”, “fidgety”, “feeling anxious”) acted as central bridging symptoms connecting communities.

### 4.1 Network structure of loneliness, anxiety and depression

Network analysis revealed that loneliness formed a separate cluster with limited connections to anxiety and depression communities, which align with those of previous studies (11). This suggests that loneliness functions as a unique psychological construct, distinct in its symptomology and mechanisms from anxiety and depression (11, 40). Unlike anxiety and depression, which are characterized by emotional, cognitive, and physical manifestations, loneliness is primarily defined by feelings of social isolation or a subjective perception of solitude (11, 40). Danneel’s study also confirmed that while loneliness, anxiety, and depression are interrelated, they follow unique trajectories, underscoring the importance of recognizing loneliness as a separate entity in mental health interventions (41). Regarding the comorbidity of anxiety and depression, previous studies found that anxiety and depression not only coexist in clinical populations but are also closely related in the general population (7, 42). This suggests that the symptoms of anxiety and depression may be mediated by shared pathophysiological mechanisms, as evidenced by their common neurobiological pathways (43).

Node predictability results showed that mean 53.5% of the variance in a node could be explained by its neighbors, suggesting a moderately interdependent symptom network. The strongest intra-community connections, such as PHQ3-PHQ4 (insomnia or hypersomnia and fatigue), GAD1-GAD2 (feeling anxious and excessive worrying), and ULS1-ULS2 (lonely and no one), align with clinical observations of shared underlying mechanisms (44–47). These findings suggest that targeting key nodes with strong intra-community connections may significantly influence the broader symptom network, thereby improving overall mental health outcomes. In contrast, inter-community connections, including GAD5-PHQ8 (fidgety-retardation), ULS6-PHQ4 (isolation-fatigue), and ULS1-GAD1 (lonely-feeling anxious), were observed to be weaker than intra-community connections, consistent with prior studies (19). Despite their relative weakness, these inter-community connections reveal specific aspects of loneliness, such as isolation (ULS6) and feeling lonely (ULS1), that are associated with depression and anxiety symptoms in nursing students. These findings underscore the importance of targeting key inter-community connections with associations to modulate symptom interactions and reduce the burden of comorbid symptoms.

### 4.2 Central symptoms of loneliness, anxiety and depression

In this study, GAD2 (excessive worrying) emerged as the most central node in the network, whereas a previous study found GAD4 (trouble relaxing) to have the highest centrality value in the network of loneliness, depression, and anxiety symptoms in adults (11). These differences reflect the specificity of the network of loneliness, anxiety and depression among Chinese nursing students. According to Cramer, GAD2 (excessive worrying) can be central within a network and affect various psychological disorders, including anxiety, depression, and associated states such as loneliness (48, 49). The rigorous demands of nursing programs, including a high workload, examination stress, performance expectations, and clinical and theoretical training, contribute to excessive worry among nursing students (50, 51). ULS6 (isolation) had a strong influence on the whole network, and Cacioppo focused on how feelings of isolation are central to the experience of loneliness, examining its impact on health and well-being and suggesting that isolation can significantly contribute to the physiological and psychological aspects of loneliness (52). Given the high levels of loneliness reported among nursing students, this highlights the need for distinct strategies to address loneliness in this population and mitigate its impact on mental health and well-being. Moreover, PHQ4 (fatigue) and PHQ2 (feeling down) were the central symptoms in the network, align to that found by Ren (7) and Owczarek (11). Gilbert and Weaver explored the relationship between poor sleep quality due to high academic demands and increased fatigue, which, in turn, affects students’ overall academic success and mental health (53). The central symptoms of GAD2 (excessive worrying), ULS6 (isolation), PHQ4 (fatigue), and PHQ2 (feeling down) in network structures suggests their potential roles as predictive indicators of severe or complex psychopathological profiles, suggesting their potential roles as pivotal points for intervention (16).

### 4.3 Bridge symptoms of loneliness, anxiety symptoms and depression

According to Fried and Cramer, bridging symptoms can both cause and sustain comorbid mental illnesses and/or syndromes (54). Consequently, bridging symptoms aid the identification of important transdiagnostic symptoms that may be used as therapeutic targets for comorbidities. In this study, PHQ8 (retardation), PHQ2 (feeling down), GAD5 (fidgety), and GAD1 (feeling anxious) emerged as the strongest bridge symptoms within the loneliness, anxiety, and depression clusters. Previous studies have also found that PHQ2 (feeling down) is a key bridging symptom in anxiety-depression network analyses (7). Bridge symptoms of PHQ8 (retardation), and GAD5 (fidgety) are part of both depressive disorder and anxiety disorder from 5th Edition of the Diagnostic and Statistical Manual of Mental Disorders (DSM-5) criteria (55). GAD1 (feeling anxious) is one of bridge nodes linking family function, anxiety and depressive symptoms (56). This is particularly noteworthy as nursing students often experience co-occurring loneliness, anxiety, and depression due to the stressors inherent in their academic and clinical environments (5, 25–27). These findings indicate that addressing PHQ8 and PHQ2 in depression may reduce the risk of progression to loneliness and anxiety, while targeting GAD5 and GAD1 in anxiety may help prevent progression to loneliness and depression. Therefore, these symptoms may be important “signs” of comorbidities and warrant attention as a focus in the assessment of nursing students. Interventions addressing “retardation”, “feeling down”, “fidgety”, and “feeling anxious” may have a broad impact in alleviating comorbidities of loneliness, anxiety, and depression among this population.

### 4.4 Comparative network analysis

The findings of this study indicate that symptoms related to anxiety (i.e., generalized anxiety and irritability) are strongly associated with sex among nursing students. Remes and colleagues comprehensively reviewed the biological and sociocultural factors that contribute to higher rates of anxiety in females compared to males (57), the latter are particularly less likely to seek help and often cope with substance use, which can influence the expression of irritability associated with anxiety (58). These findings highlight the importance of assessing the gender among nursing students’ sex when they show mental symptoms and underline the need for sex-specific approaches in mental health treatment and research.

## 5 Limitations

First, the cross-sectional design restricted our ability to infer causality among the symptoms of loneliness, anxiety, and depression. Although our findings lay a strong foundation for future studies to build upon focused causal hypotheses, longitudinal research is necessary to establish definitive causal relationships. Second, logistical constraints (e.g., time constraints, access issues, and participant burden) limited our exploration of the various potential risks and protective factors that might influence the interplay among loneliness, anxiety, and depression. Important variables, such as family, university, and social factors, were not assessed. Future studies should aim to incorporate these factors to provide a more comprehensive understanding. Additionally, the reliance on self-reported measures introduces the possibility of recall bias or social desirability effects that could skew the results. Efforts to include objective measures in future studies could help to mitigate these biases. Finally, the geographical scope of our study was confined to nursing students in southern China, which limits the generalizability of our findings to other regions or countries. Subsequent research should consider more diverse demographics to enhance the global applicability of these results. Future research should also delve deeper into the mechanisms by which these central symptoms interact and influence disease progression, potentially unveiling more effective customized therapeutic approaches.

## 6 Conclusions

This study showed that loneliness remains a distinct and relatively independent experience, whereas depression and anxiety symptoms are significantly interconnected among nursing students. Central and bridge symptoms have been identified as crucial connectors across these conditions, suggesting their potential as targets for therapeutic intervention. Addressing these core symptoms can effectively reduce the overall impact of mental health problems. Future research should focus on these relationships over time to develop more precise targeted interventions.

## Data Availability

The data that support the findings of this study are available from the corresponding author upon reasonable request.
