# Supplementary material for: Exploring the interconnections of loneliness, anxiety, and depression among nursing students: a network analysis approach
Source: Front Psychiatry. 2025 Feb 17;16:1537935. doi: 10.3389/fpsyt.2025.1537935 (PMC11873105; doi:10.3389/fpsyt.2025.1537935)
Supplement: Supplementary file 1 [file Supplementaryfile1.docx]

Supplementary Material

# Supplementary Figures and Tables

For more information on Supplementary Material and for details on the different file types accepted, please see [here](https://www.frontiersin.org/guidelines/author-guidelines#supplementary-material).

## Supplementary Figures


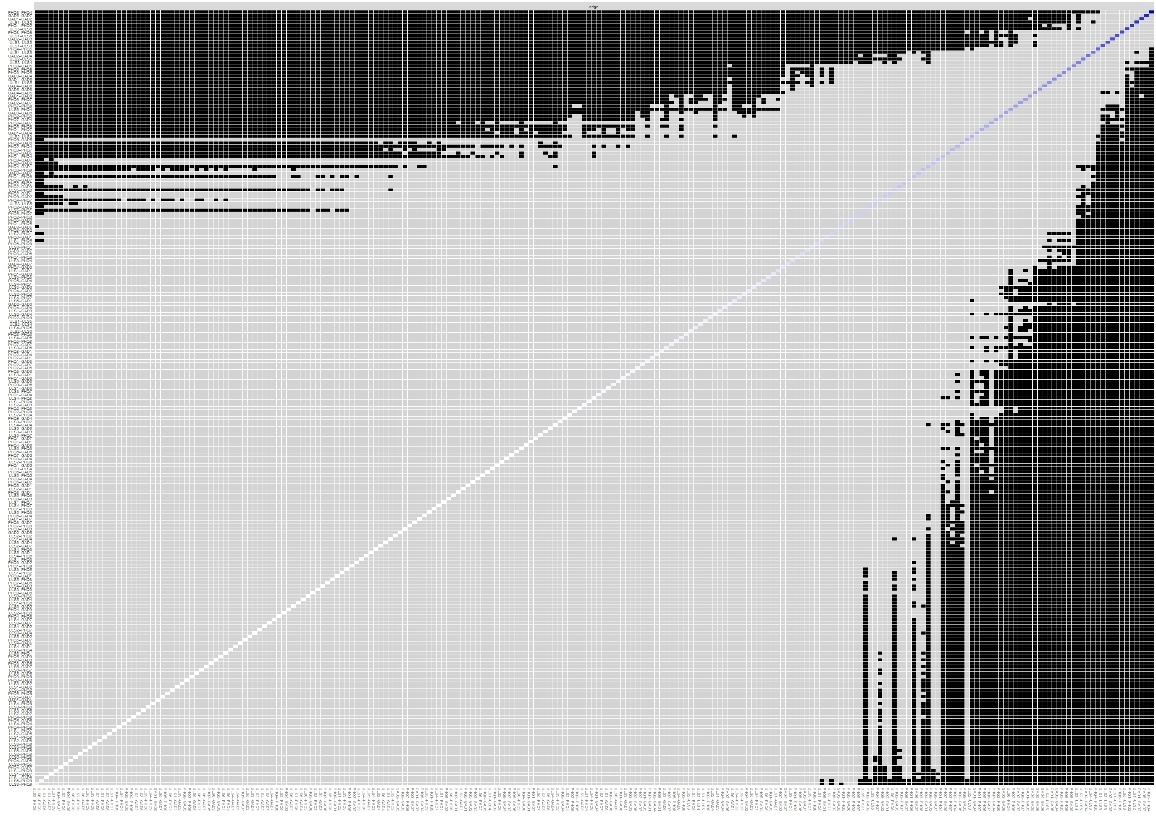


**Supplementary Figure 1.** Bootstrapped difference test for edge weights. Note: Gray boxes indicate no significant differences between edges, black boxes indicate significant differences between edges. The diagonal lines indicate the strength of the edge weights, from red (representing negative associations) to dark blue (representing positive associations).


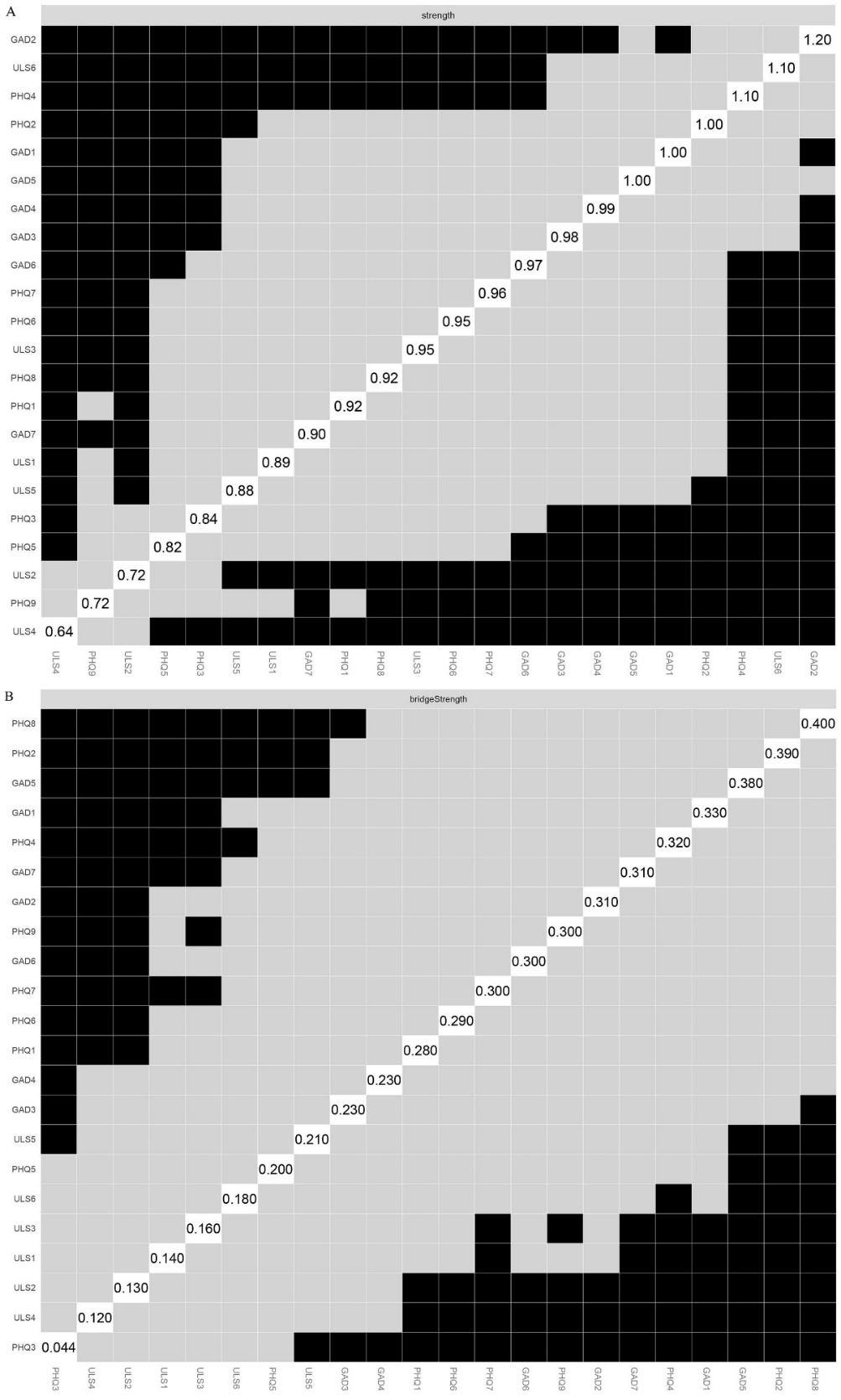


**Supplementary Fig.2**. A: Nonparametric bootstrapped difference test for strength. B: Nonparametric bootstrapped difference test for bridge strength. Note: Gray boxes indicate no difference between nodes and black boxes indicate significant differences (α = 0.05). Values on the diagonal represent intensity values for each nod

## Supplementary Tables

| Supplementary Table1. Description of the analytical approach | |
| --- | --- |
| Analytical approach | |
| Central symptom | To explore the importance of individual symptoms in the network, four centrality indices, strength, closeness, betweenness, and expected influence, were calculated. Higher values of the indices mean higher importance in the network (Epskamp et al., 2012). Strength refers to the sum of absolute edge weights connected to a node. The closeness means the inverse of the length of all shortest paths from one node to all other nodes. Betweenness is the importance of a symptom as a ‘connector’ with other symptoms based on how often the node lies on all the shortest paths between other nodes (Opsahl et al., 2010). The expected influence is defined as the sum of the values of all edges connected to a particular node. |
| Bridging symptoms | To explore bridging symptoms that play an important role in connecting two different communities with symptoms of loneliness, depression, and anxiety, the bridging function of R-package network tools was used. The bridge centrality indices used in this study include bridge strength, bridge closeness, bridge betweenness, and bridge expected influence. A higher indices value indicates a higher probability of spreading the symptom to other communities (Jones et al., 2021). |
| Network stability and accuracy | Three analyses were conducted in this study using R-package boonet packages to assess the stability and accuracy of the network model. First, the correlation stability (CS) coefficients were calculated based on the case-drop bootstrap method (1000 iterations) (Popovic et al., 2015). The CS coefficient should be above 0.25 (acceptable level) and preferably above 0.5 (Epskamp et al., 2018a). Second, the accuracy of the edge weights was estimated based on a nonparametric bootstrap method (1000 iterations). The narrower the 95% CI, the higher the accuracy of the edge weights (Fruchterman and Reingold, 1991). Finally, bootstrapped difference tests were utilized to evaluate the edge weights, strengths, bridge strengths, closeness and expected influence to test whether there was significant difference between the two edge weights, the strengths, closeness and expected influence of the two nodes, and the bridge strengths of the two nodes. |
| Comparative network analysis | This study compared differences in networks across gender and grades using the Network Comparison Test (NCT) in the Network Comparison Test package of the R software (Lai et al., 2020). The method was sampled with subsamples of 1000 permutations (i.e., female vs. male, lower vs. upper grades of participants) and aimed to assess the global network strength and network structure between two networks (Zhang et al., 2020). In addition, multiple comparisons using Holm-Bonferroni correlations were conducted to assess the strength of each edge between the two networks. |

**Supplementary Table 2. Edge weight matrix**

|  | **ULS1** | **ULS2** | **ULS3** | **ULS4** | **ULS5** | **ULS6** | **PHQ1** | **PHQ2** | **PHQ3** | **PHQ4** | **PHQ5** | **PHQ6** | **PHQ7** | **PHQ8** | **PHQ9** | **GAD1** | **GAD2** | **GAD3** | **GAD4** | **GAD5** | **GAD6** | **GAD7** |
| --- | --- | --- | --- | --- | --- | --- | --- | --- | --- | --- | --- | --- | --- | --- | --- | --- | --- | --- | --- | --- | --- | --- |
| **ULS1** | 0.000 |  |  |  |  |  |  |  |  |  |  |  |  |  |  |  |  |  |  |  |  |  |
| **ULS2** | 0.273 | 0.000 |  |  |  |  |  |  |  |  |  |  |  |  |  |  |  |  |  |  |  |  |
| **ULS3** | 0.224 | 0.103 | 0.000 |  |  |  |  |  |  |  |  |  |  |  |  |  |  |  |  |  |  |  |
| **ULS4** | 0.000 | 0.024 | 0.164 | 0.000 |  |  |  |  |  |  |  |  |  |  |  |  |  |  |  |  |  |  |
| **ULS5** | 0.024 | 0.175 | 0.067 | 0.148 | 0.000 |  |  |  |  |  |  |  |  |  |  |  |  |  |  |  |  |  |
| **ULS6** | 0.232 | 0.023 | 0.229 | 0.182 | 0.259 | 0.000 |  |  |  |  |  |  |  |  |  |  |  |  |  |  |  |  |
| **PHQ1** | 0.000 | 0.057 | 0.048 | 0.031 | 0.066 | 0.000 | 0.000 |  |  |  |  |  |  |  |  |  |  |  |  |  |  |  |
| **PHQ2** | 0.000 | 0.000 | 0.005 | 0.030 | 0.000 | 0.000 | 0.266 | 0.000 |  |  |  |  |  |  |  |  |  |  |  |  |  |  |
| **PHQ3** | 0.000 | 0.030 | 0.000 | 0.000 | 0.000 | 0.001 | 0.046 | 0.062 | 0.000 |  |  |  |  |  |  |  |  |  |  |  |  |  |
| **PHQ4** | 0.008 | 0.005 | 0.043 | 0.000 | 0.000 | 0.124 | 0.084 | 0.095 | 0.332 | 0.000 |  |  |  |  |  |  |  |  |  |  |  |  |
| **PHQ5** | 0.053 | 0.000 | 0.000 | 0.024 | 0.034 | 0.000 | 0.059 | 0.007 | 0.155 | 0.201 | 0.000 |  |  |  |  |  |  |  |  |  |  |  |
| **PHQ6** | 0.000 | 0.000 | 0.000 | 0.000 | 0.070 | 0.000 | 0.081 | 0.118 | 0.022 | 0.000 | 0.109 | 0.000 |  |  |  |  |  |  |  |  |  |  |
| **PHQ7** | 0.000 | 0.000 | 0.010 | 0.000 | 0.003 | 0.000 | 0.108 | 0.074 | 0.093 | 0.067 | 0.064 | 0.131 | 0.000 |  |  |  |  |  |  |  |  |  |
| **PHQ8** | 0.000 | 0.000 | 0.000 | 0.008 | 0.000 | 0.000 | 0.000 | 0.006 | 0.082 | 0.000 | 0.020 | 0.052 | 0.126 | 0.000 |  |  |  |  |  |  |  |  |
| **PHQ9** | 0.000 | 0.000 | -0.020 | 0.000 | 0.000 | -0.013 | 0.000 | 0.024 | 0.000 | 0.000 | 0.000 | 0.156 | 0.000 | 0.240 | 0.000 |  |  |  |  |  |  |  |
| **GAD1** | 0.040 | 0.000 | 0.013 | 0.000 | 0.000 | 0.029 | 0.002 | 0.070 | 0.000 | 0.082 | 0.000 | 0.055 | 0.017 | 0.018 | 0.000 | 0.000 |  |  |  |  |  |  |
| **GAD2** | 0.000 | 0.000 | 0.000 | 0.000 | 0.000 | 0.000 | 0.000 | 0.066 | 0.000 | 0.030 | 0.000 | 0.083 | 0.000 | 0.058 | 0.069 | 0.281 | 0.000 |  |  |  |  |  |
| **GAD3** | 0.027 | 0.008 | 0.003 | 0.000 | 0.031 | 0.000 | 0.034 | 0.074 | 0.013 | 0.000 | 0.000 | 0.000 | 0.040 | 0.000 | 0.000 | 0.133 | 0.230 | 0.000 |  |  |  |  |
| **GAD4** | 0.000 | 0.026 | 0.000 | 0.004 | 0.000 | 0.000 | 0.008 | 0.018 | 0.000 | 0.027 | 0.032 | 0.000 | 0.112 | 0.000 | 0.005 | 0.104 | 0.182 | 0.155 | 0.000 |  |  |  |
| **GAD5** | 0.000 | 0.000 | 0.000 | 0.000 | 0.000 | 0.000 | 0.016 | 0.015 | 0.000 | 0.000 | 0.000 | 0.000 | 0.093 | 0.156 | 0.098 | 0.000 | 0.000 | 0.059 | 0.133 | 0.000 |  |  |
| **GAD6** | 0.011 | 0.000 | 0.020 | 0.021 | 0.004 | 0.012 | 0.012 | 0.097 | 0.000 | 0.001 | 0.062 | 0.000 | 0.000 | 0.047 | 0.012 | 0.153 | 0.029 | 0.124 | 0.144 | 0.139 | 0.000 |  |
| **GAD7** | 0.000 | 0.000 | 0.000 | 0.000 | 0.000 | 0.000 | 0.003 | 0.015 | 0.000 | 0.000 | 0.000 | 0.078 | 0.020 | 0.110 | 0.084 | 0.000 | 0.130 | 0.047 | 0.043 | 0.287 | 0.081 | 0.000 |
| Note: ULS: UCLA Loneliness Scale; PHQ: Patient Health Questionnaire; GAD: General Anxiety Disorder. | | | | | | | | | | | | | | | | | | | | | | |

**Supplementary Table 3. Centrality estimates of nodes in the network (n = 888)**

| **Items** | **Strength** | **Betweenness** | **Closeness** | **EI** | **Pre** | **Bridge**  **strength** | **Bridge**  **betweenness** | **Bridge**  **closeness** | **BEI** |
| --- | --- | --- | --- | --- | --- | --- | --- | --- | --- |
| ULS1 | .891 | 7 | .002 | .900 | .465 | .138 | 5 | .039 | .138 |
| ULS2 | .723 | 1 | .002 | .891 | .309 | .125 | 1 | .037 | .125 |
| ULS3 | .949 | 0 | .002 | .723 | .470 | .163 | 0 | .039 | .122 |
| ULS4 | .637 | 0 | .002 | .909 | .294 | .119 | 0 | .038 | .119 |
| ULS5 | .881 | 20 | .003 | .637 | .426 | .208 | 19 | .045 | .208 |
| ULS6 | 1.104 | 54 | .003 | .881 | .551 | .179 | 51 | .046 | .153 |
| PHQ1 | .922 | 8 | .003 | 1.079 | .536 | .278 | 7 | .053 | .278 |
| PHQ2 | 1.043 | 10 | .003 | .922 | .592 | .390 | 8 | .055 | .390 |
| PHQ3 | .838 | 17 | .003 | 1.043 | .505 | .044 | 13 | .056 | .044 |
| PHQ4 | 1.100 | 61 | .003 | .838 | .599 | .321 | 57 | .063 | .321 |
| PHQ5 | .819 | 2 | .003 | 1.100 | .443 | .204 | 0 | .052 | .204 |
| PHQ6 | .955 | 25 | .003 | .819 | .528 | .286 | 21 | .057 | .286 |
| PHQ7 | .959 | 8 | .003 | .955 | .553 | .296 | 7 | .053 | .296 |
| PHQ8 | .924 | 10 | .003 | .959 | .563 | .397 | 7 | .049 | .397 |
| PHQ9 | .721 | 4 | .003 | .924 | .458 | .301 | 2 | .049 | .234 |
| GAD1 | .997 | 27 | .003 | .655 | .629 | .326 | 26 | .051 | .326 |
| GAD2 | 1.158 | 15 | .003 | .997 | .697 | .306 | 12 | .048 | .306 |
| GAD3 | .977 | 0 | .002 | 1.158 | .638 | .230 | 0 | .042 | .230 |
| GAD4 | .994 | 6 | .002 | .977 | .642 | .233 | 5 | .042 | .233 |
| GAD5 | .997 | 11 | .002 | .994 | .641 | .378 | 9 | .044 | .378 |
| GAD6 | .968 | 8 | .003 | .997 | .615 | .298 | 7 | .046 | .298 |
| GAD7 | .891 | 5 | .002 | .968 | .624 | .311 | 4 | .042 | .311 |

Note: ULS: UCLA Loneliness Scale; PHQ: Patient Health Questionnaire; GAD: General Anxiety Disorder; EI: Expected Influence; Pre: Predictability; BEI: Bridge Expected Influence.
